# Margarita Dermatitis Revisited: A Case Report and Literature Review of Lime-Associated Phytophotodermatitis

**DOI:** 10.7759/cureus.116249

**Published:** 2026-09-14

**Authors:** Philip R Cohen, Benjamin L Ramsay

**Affiliations:** 1 Dermatology, University of California Davis Medical Center, Sacramento, USA; 2 Dermatology, Touro University California College of Osteopathic Medicine, Vallejo, USA; 3 Biomedical Engineering, Johns Hopkins University, Baltimore, USA

**Keywords:** dermatitis, hyperpigmentation, lime, margarita, patch, phototoxic, phytophotodermatitis, pigmentation, sun-exposed, ultraviolet

## Abstract

Phytophotodermatitis is a phototoxic skin reaction that occurs after topical contact with certain plant substances followed by exposure of the affected skin area to the sun. A unique type of phytophotodermatitis is margarita dermatitis. This results from contact with either lime peel or lime juice, or both, with the skin and subsequent exposure to ultraviolet A radiation to the lime-contacted skin. Other variants of lime-associated, alcoholic beverage-related phytophotodermatitis are mojito dermatitis and Mexican beer dermatitis. Lime-induced phytophotodermatitis typically results in the acute onset of symptoms and lesions, such as painful and occasionally pruritic erythema and blisters, after the skin that has been in contact with the lime is exposed to the sun. As the initial lesions resolve, postinflammatory hyperpigmentation occurs, which often eventually improves or completely resolves. In many circumstances, the patient is unaware that not only the contact with lime and the skin but also the subsequent sun exposure were related to the development of their new cutaneous dermatosis; indeed, the clinician evaluating these individuals needs to have a high level of suspicion that the patient’s new lesions may be a phytophotodermatitis. Often, the person with acute manifestations or chronic hyperpigmentation associated with lime-related phytophotodermatitis is initially diagnosed with an incorrect condition by the medical personnel who are performing the evaluation. The differential diagnosis can include primary bullous conditions, atypical bruising or purpura, other types of dermatitis, infections, drug reactions, pigmentary disorders, and trauma such as child abuse when a pediatric patient presents for assessment. Less commonly, lime-related phytophotodermatitis can present with asymptomatic delayed hyperpigmentation. A 28-year-old man is described whose margarita dermatitis presented with asymptomatic pigmentation on his hands three days after exposure to limes and sunlight. Including this patient, lime-associated asymptomatic hyperpigmentation phytophotodermatitis has been reported in 14 individuals. The pigment that was most common on the dorsal hands (11 patients) and acquired while preparing a beverage or meal for consumption that included lime was the most common exposure to the lime; the pigment occurred on the back in the remaining three patients. The pigment appeared from two days up to three to seven weeks (median, seven days) after exposure to the lime. *MA*garita *DE*rmatitis by a *S*liced/*S*queezed *L*ime-*A*ssociated *P*hytophotodermatitis (*MADE* by a *SLAP*) is a new acrostic that we introduce to refer to this condition in an individual with phytophotodermatitis caused by lime-related margarita dermatitis.

## Introduction

Phytophotodermatitis is a nonimmunologic cutaneous reaction that results from the sequential contact of certain plant substances with the skin and exposure of that area to the sun. It typically presents as painful erythema with or without vesicles and blisters at the sites of contact. Hyperpigmentation usually develops as the acute reaction resolves [1-3].

The affected individual may not readily associate the initial plant exposure and subsequent sun exposure as the etiology of their cutaneous phytophotodermatitis lesions. This may allow several conditions to be considered in the clinical differential diagnosis of the patient’s skin condition. Therefore, the clinician must entertain the possibility of phytophotodermatitis in order to seek a consistent medical history from the patient that can establish the suspected diagnosis [1,2,4-6].

Margarita dermatitis is a unique variant of phytophotodermatitis. A margarita is an alcoholic beverage consisting of tequila, orange liqueur, and lime juice. After contact of the skin with either the lime peel or the lime juice, or both, during preparation of the beverage, the phytophotodermatitis develops when the lime-contacted skin sites are exposed to sunlight. Hence, when the etiology of phytophotodermatitis occurs during the preparation of a margarita cocktail, the resulting lime-induced skin condition has sometimes been referred to as margarita dermatitis [1,3,6-9].

We present a man who developed pigmentation on his dorsal hands and digits after cutting limes for margarita preparation during a bachelor party. His manifestation of lime-associated phytophotodermatitis was unique in that it presented with asymptomatic hyperpigmentation on his hands three days after the sequential contact of his skin with the citrus fruit followed by sun exposure. The features of phytophotodermatitis related to limes are summarized, and the characteristics of 14 individuals, including the reported patient, with asymptomatic delayed pigmentation as their presentation of lime-induced phytophotodermatitis are reviewed [6,10-13].

## Case presentation

A 28-year-old man was attending a men’s-only reception for a future groom (a bachelor’s party) in Holland, Michigan, on Day 1. He had been assigned to assist in the preparation of the margaritas that were to be consumed later that day. Limes had been purchased to be used in the beverage (Figure 1).

**Figure 1:**
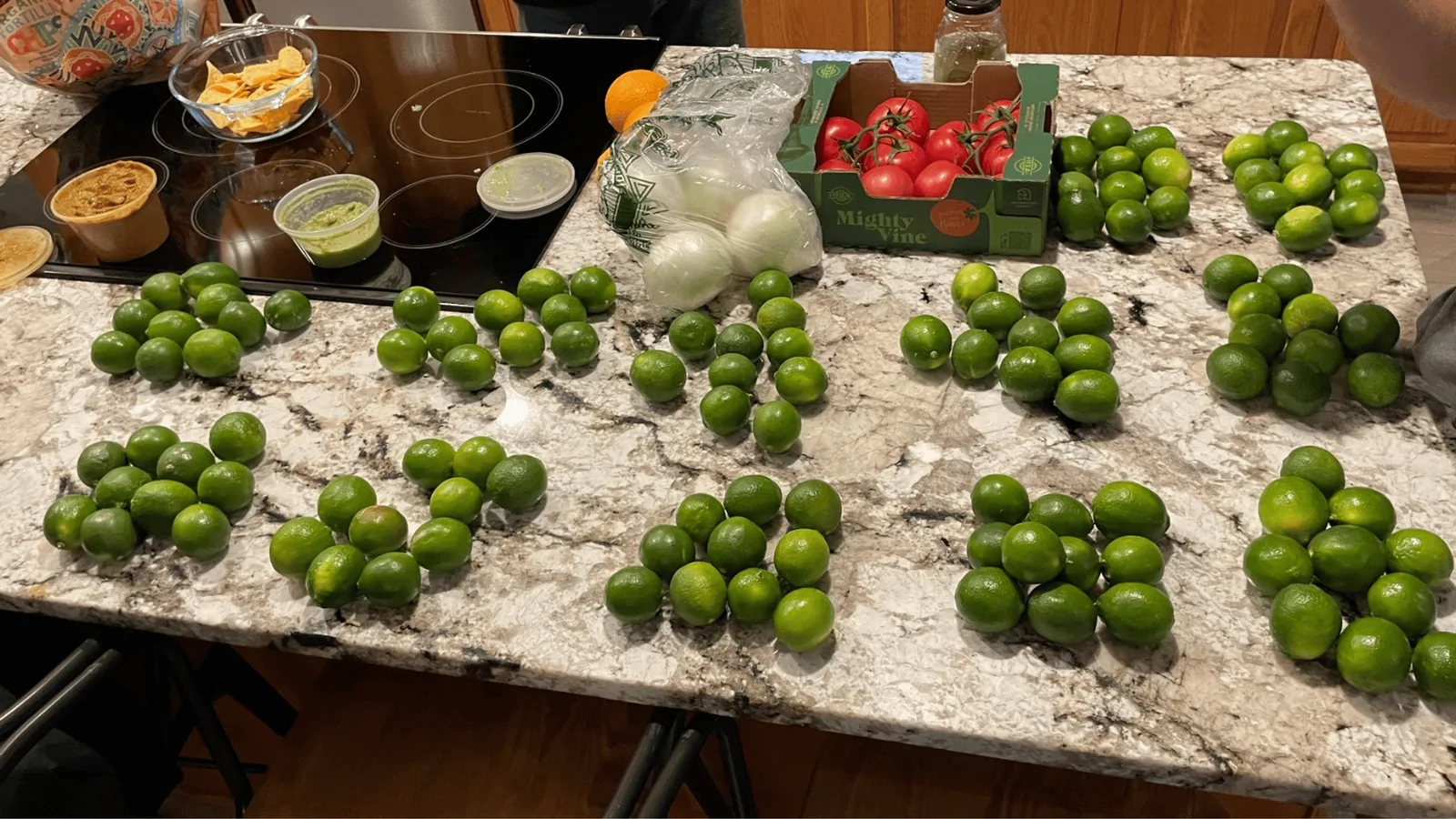
Limes on the table to be used in preparation for margaritas There a total of 120 limes (arranged in groups of 10 limes) on the table. Some of the limes were squeezed to collect fresh lime juice.

The right-handed man began cutting each of the limes in half at 11:30 AM; he was not wearing gloves. He used a hand-operated pressing machine to squeeze the juice from each lime. By noon, he had collected a total of 250 milliliters of lime juice, which was approximately 12.5 milliliters per lime (Figure 2).

**Figure 2:**
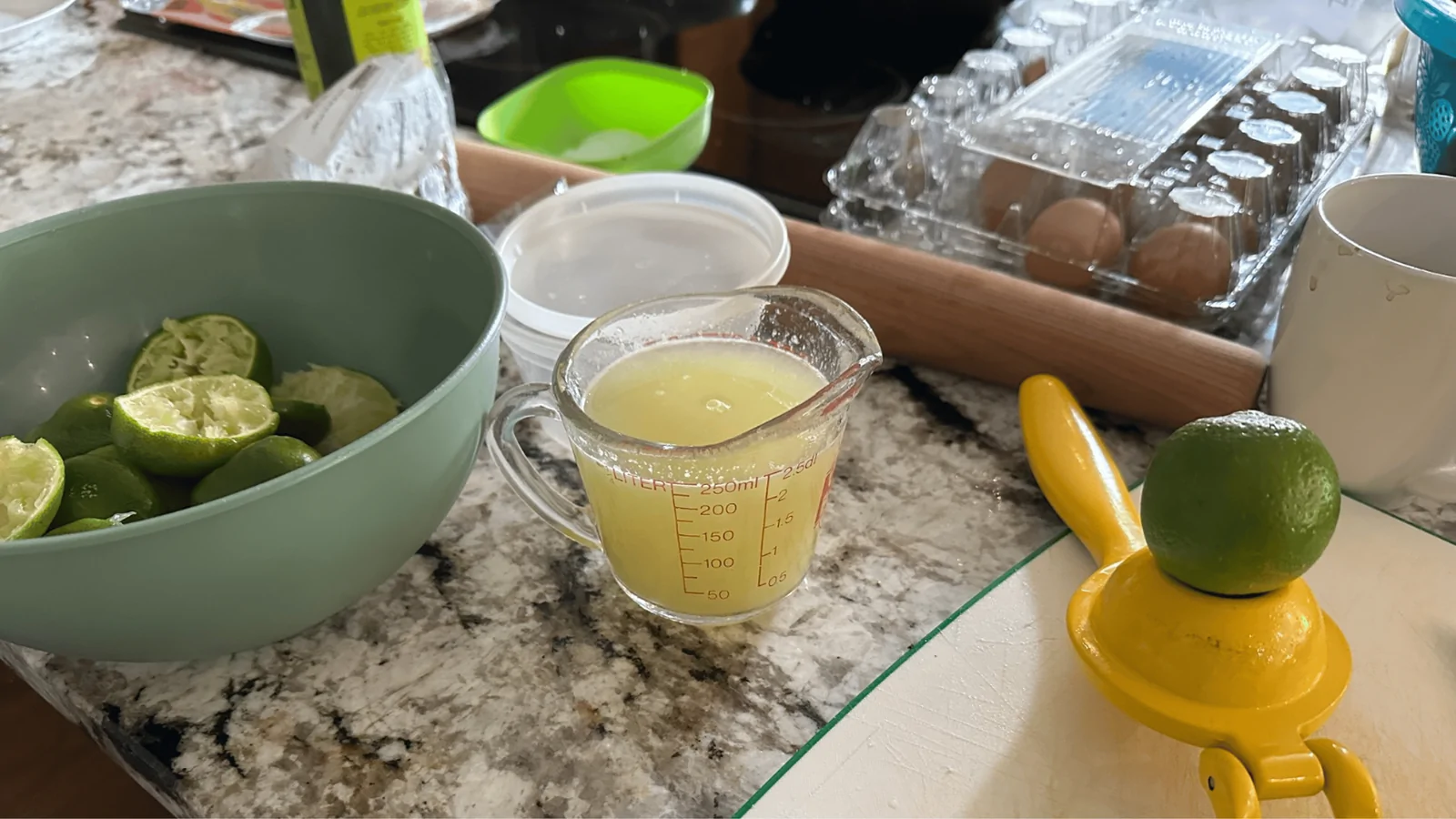
Collection of lime juice A little more than 250 milliliters of fresh lime juice was collected after squeezing 20 limes. A hand-operated pressor was used to obtain approximately 12.5 milliliters of juice from each lime. Some of the discarded limes are in the blue bowl.

It was a beautiful, mild, sunny day, with occasional periods of cloud overcast, in late Spring. The outside temperature was 72 degrees Fahrenheit. He was outside in a short-sleeved shirt with his distal arms and hands exposed for more than four hours participating in miniature golf, go-karting, fluid hydration, and limeball.

During his time outside, he did not experience any pruritus or tenderness on his hands. Later that evening and the next day, there was no erythema. However, three days later, he noticed the new appearance of asymptomatic brown patches on his hands. The areas of hyperpigmentation became darker.

Photographs of his dorsal hands were taken about three weeks later (Figure 3). They showed hyperpigmentation on the dorsal left and right hands. The skin near some of the metacarpophalangeal joints and proximal fingers also demonstrated hyperpigmentation.

**Figure 3:**
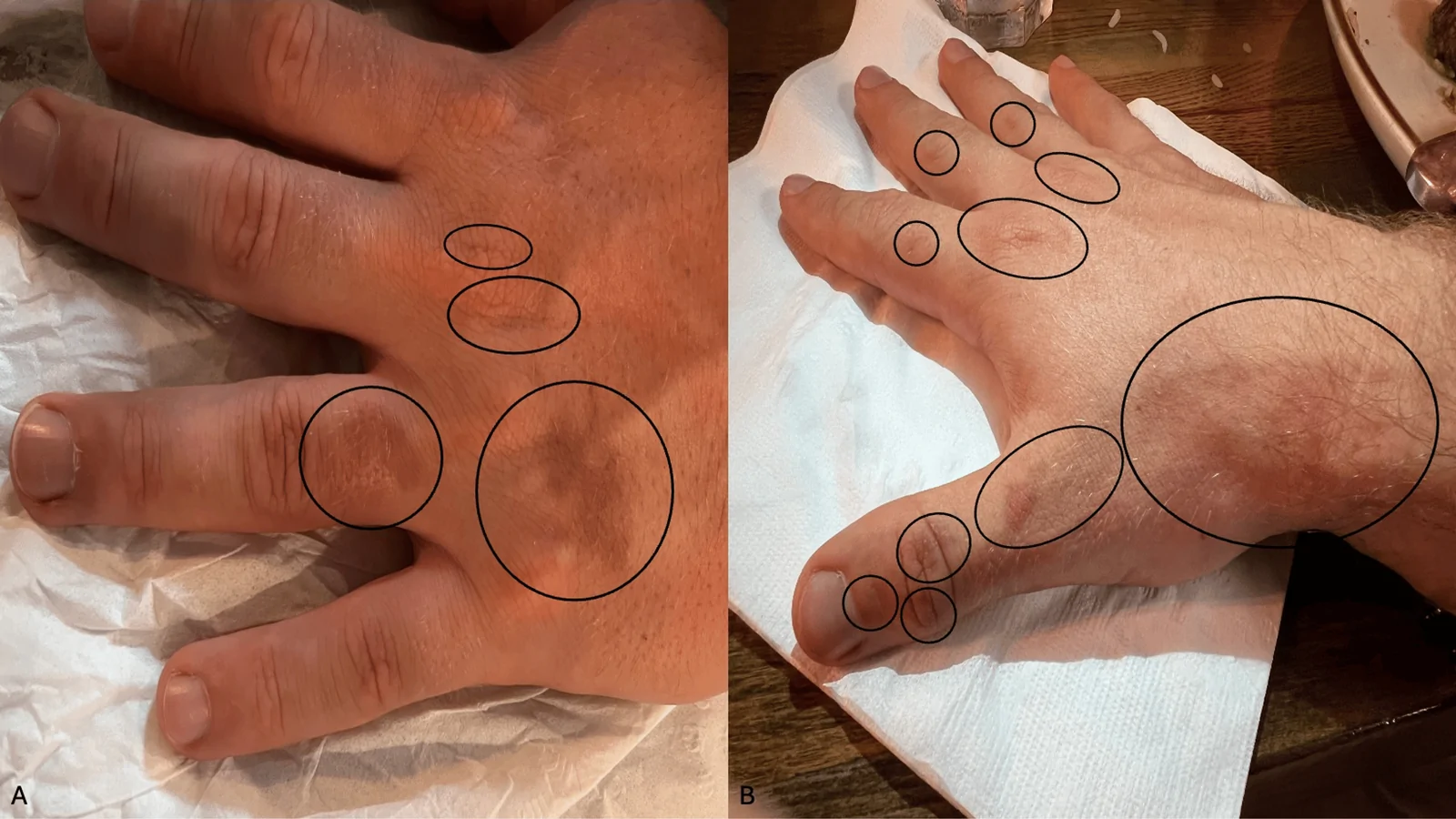
Phytophotodermatits on the dorsal hands of a 28-year-old man presenting as asymptomatic hyperpigmentation on both of his hands After slicing and using a machine to squeeze 20 limes on a Saturday morning, a man spent several hours outside on a sunny afternoon. Three days later, on the following Tuesday, the man noted darkening on the dorsal aspect of both his left (A) and right (B) hands. The black ovals surround some of the areas of new pigment on his hands.

About four weeks thereafter, additional photographs of his left hand (Figures 4, 5) and right hand (Figures 6, 7) were taken. The findings were similar to those observed approximately a month earlier. The hyperpigmentation was beginning to lighten.

**Figure 4:**
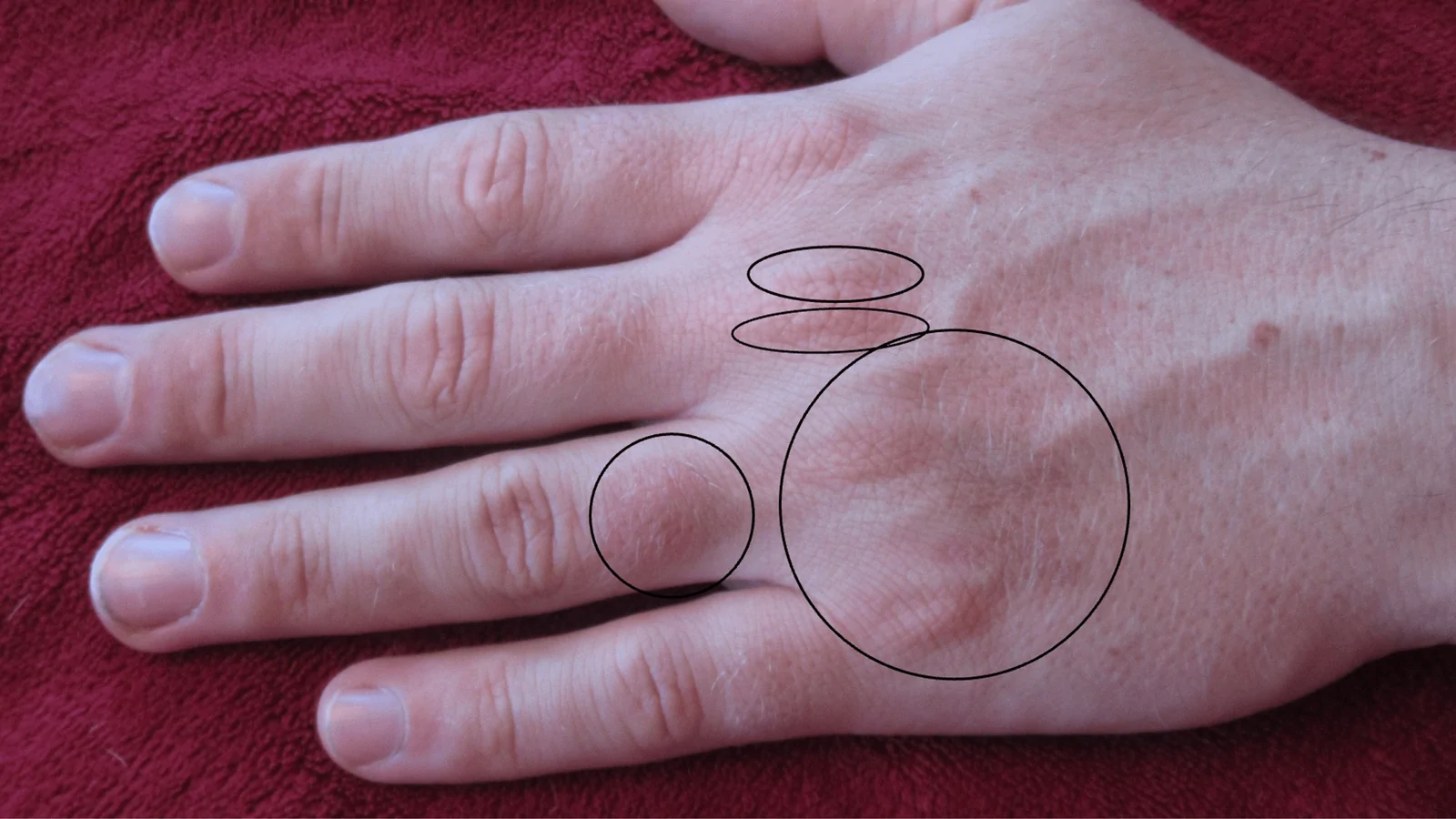
Margarita dermatitis on the left hand presenting as asymptomatic hyperpigmentation Phytophotodermatitis presented as dark pigment (demonstrated by the black ovals surrounding some of the areas of hyperpigmentation) on the back of a man’s hand; brown discoloration is present on skin overlying the left third, fourth, and fifth metacarpal-phalangeal joints and proximal left fourth digit.

**Figure 5:**
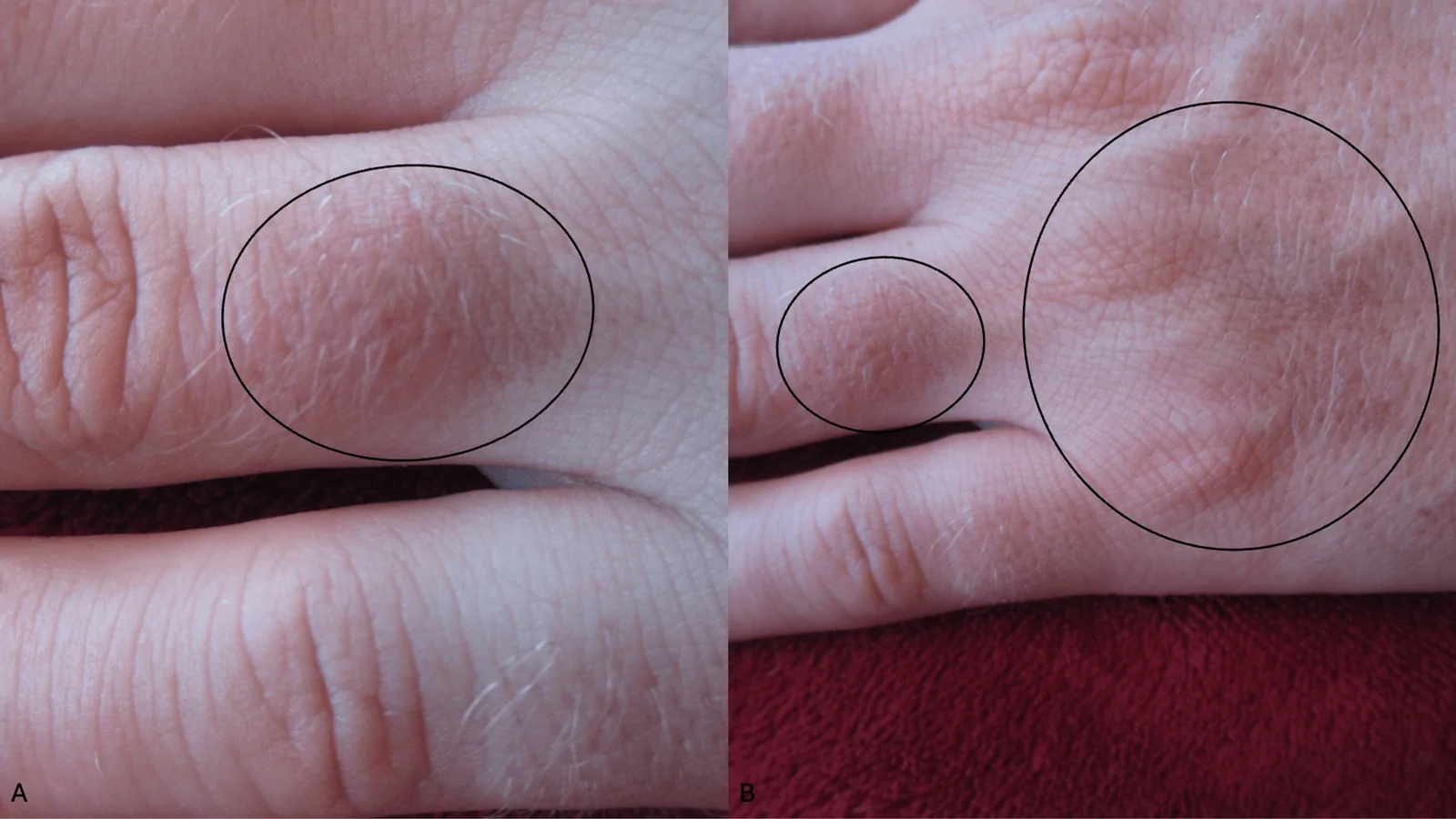
Closer views of lime-associated phytophotodermatitis on the left hand Margarita dermatitis presenting as asymptomatic hyperpigmentation (demonstrated by the dark areas surrounded by the black ovals). A closer view of the brown discoloration on the man’s proximal left fourth digit (A) and a more distant view of the dorsal left hand showing pigment on the area surrounding the fourth and metacarpal-phalangeal joints and proximal left fourth digit (B).

**Figure 6:**
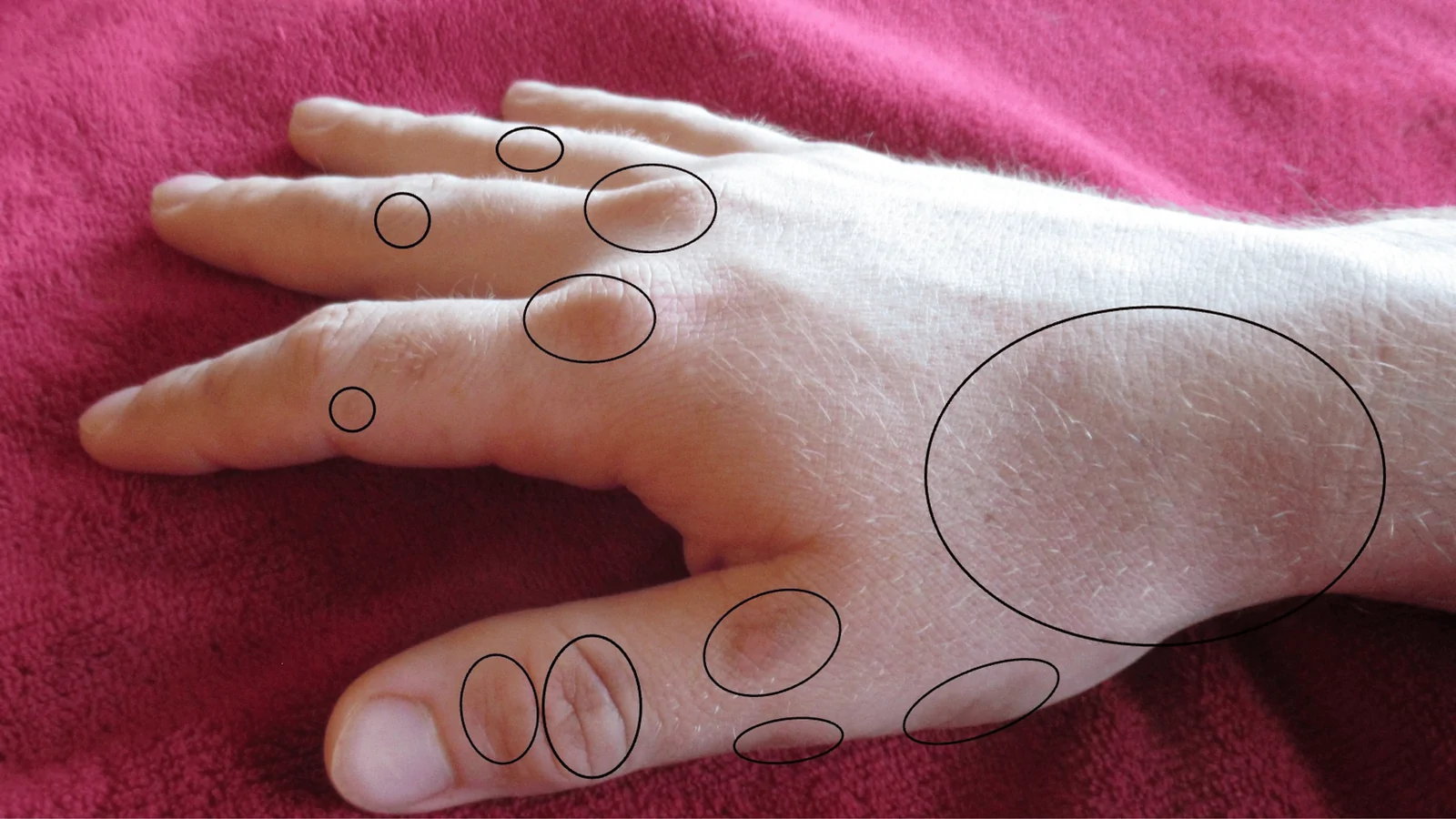
Asymptomatic pigmented patches are the manifestation of margarita dermatitis on the right hand The black ovals surround several of the dark patches caused by the lime-associated phytophotodermatitis on the man’s dorsal right hand. Brown pigmentation is present not only on the right thumb and three adjacent digits but also the skin overlying the second and third metacarpal-phalangeal joints.

**Figure 7:**
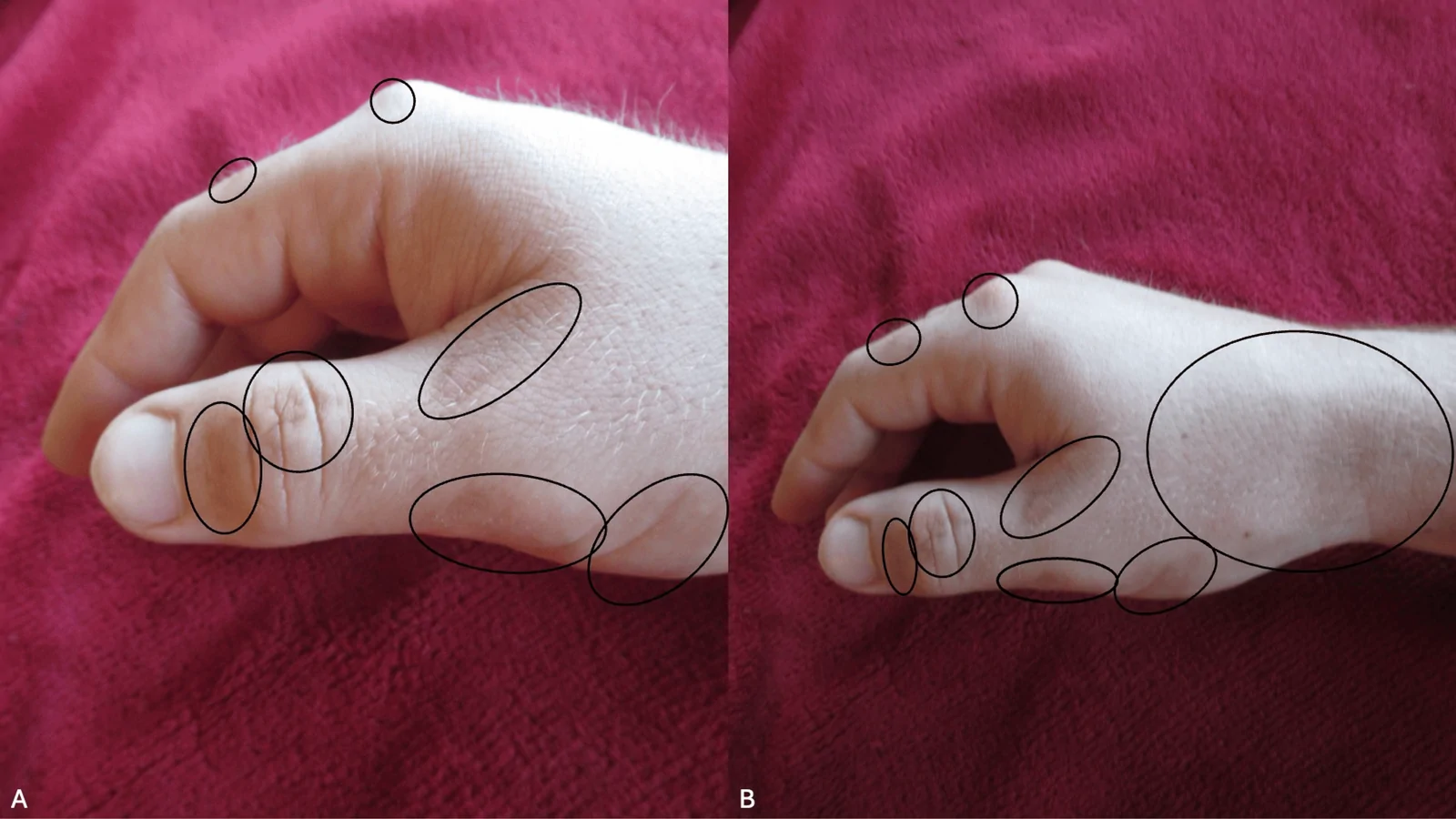
Closer views of lime-related phytophotodermatitis on the right hand Asymptomatic hyperpigmentation (demonstrated by the dark areas surrounded by the black ovals) as the presenting manifestation of Margarita dermatitis on a man’s right hand. A closer view (A) and more distant view (B) showing pigment areas on the man’s proximal right thumb and second digit.

Correlation of the clinical history and morphologic presentation of the skin lesions established a diagnosis of lime-associated phytophotodermatitis (margarita dermatitis). The man recalled not only the lime peel rubbing on his hands but also the lime juice splashing on his hands during the cutting and squeezing of the fruit. The unusual feature in his situation is that he did not experience any symptoms or lesions during or after sun exposure and that he only developed painless hyperpigmentation as a clinical sequela of his phytophotodermatitis.

The hyperpigmentation completely faded without any medication during the subsequent four months. It has not recurred.

## Discussion

Cutaneous ultraviolet-related photosensitivity can be associated with exogenous agents. The sequential exposure of an individual to the drug and sunlight is necessary for the reaction to occur. Exogenous photosensitivity reactions include photoallergic reactions or phototoxic reactions [8,14,15].

Photoallergic reactions have a low incidence of occurrence and only require a small amount of the associated agent for photosensitivity. They are a type of delayed hypersensitivity reaction that requires prior immunization with the associated photosensitizer and typically occur 24 hours or more after subsequent exposure to the agent and ultraviolet radiation. Their morphologic appearance is a dermatitis (and they either have an acute eczematous, subacute, or chronic presentation), and they initially occur in sun-exposed skin and may subsequently extend to skin that has not been exposed to the sun; postinflammatory hyperpigmentation may develop during the resolution of the condition [8,14,15].

Phototoxic reactions are common skin reactions that typically occur within minutes to hours after exposure to the photosensitizer and sunlight; they can potentially be observed in any individual who receives a sufficient quantity of the causative medication followed by sufficient exposure to ultraviolet radiation and/or visible light. They do not require immunization with the eliciting agent and can occur after the initial exposure to the drug. Clinically, phototoxic reactions are usually dose-related and present as an exaggerated sunburn only on the sun-exposed skin; subsequent pigmentary changes are frequently observed. The reactions are usually associated with a systemically received medication; yet they can develop after topical exposure to the causative agent [8,14-16].

Phytophotodermatitis is a phototoxic reaction. It can result after the skin of an individual has come in contact with plants that contain a phototoxic substance such as a furanocoumarin. When the person is subsequently exposed to sunlight (and particularly ultraviolet A radiation from 320 to 400 nanometers), symptomatic erythema appears [2-4,9,13,16-21].

The affected individual usually experiences pain as a lesion-associated symptom; however, in some patients, the lesions are pruritic. Vesicles and blisters may occur. Subsequently, as the acute lesions resolve, post-inflammatory hyperpigmentation often develops. Less commonly, in some patients (such as the man in this report), phytophotodermatitis manifests only as asymptomatic pigmentation of the skin [2-4,9,13,16-21].

Several families of plants have been associated with phytophotodermatitis (Table 1) [4,9,10,22]. Celery is a common culprit [13]. However, limes can frequently cause the condition; phytophotodermatitis can also occur after contact with other citrus fruits such as lemons, grapefruits, or oranges [7,10,23]. The condition develops following contact not only with the peel of the fruit but also with the juice from the skin and subsequent exposure to sunlight [5-7,9,13,23-27].

**Table 1:** Common plants containing furocoumarins ^a^The current table is a modification of the orginal table that has been republished with permission [9].

Family	Common examples	References
Brassicaceae	Mustard	[4,9,10]^a^
Fabaceae	Bavachi, scurf-pea	[9]^a^
Moraceae	Fig	[4,9,10,22]^a^
Pimpinella	Anise seed	[9]^a^
Psoralea	Bavachee	[9]^a^
Ranunculaceae	Buttercup	[4,9,10]^a^
Rutacreae	Bergamot, bergot, bitter orange, gas plant, lemons, limes, Persian lime, rue	[4,9,10]^a^
Umbelliferae	Angelica, carrots (garden and wild), celery, dill, fennel, hogweed, parsnip, parsley	[4,9,10]^a^

Many of the affected individuals with lime-related phytophotodermatitis experience an acute onset of symptoms shortly after sun exposure. Pain is the most common symptom. However, some individuals present with pruritus; occasionally the lesions both hurt and itch [2,3,7,9,11,13,18,24,28].

The sun-exposed skin that has contacted the lime peel or juice usually develops erythema and may blister. The lesions may have a dripping, yet non-specific pattern; although their arrangement can be linear and streaky, they are not dermatomal. Subsequently, after the skin lesions associated with the acute phase resolve, there is pigmentation at the location of the prior lesions (Figures 8, 9). Eventually, the hyperpigmentation typically fades spontaneously [3,5,9,18,24,26,28].

**Figure 8:**
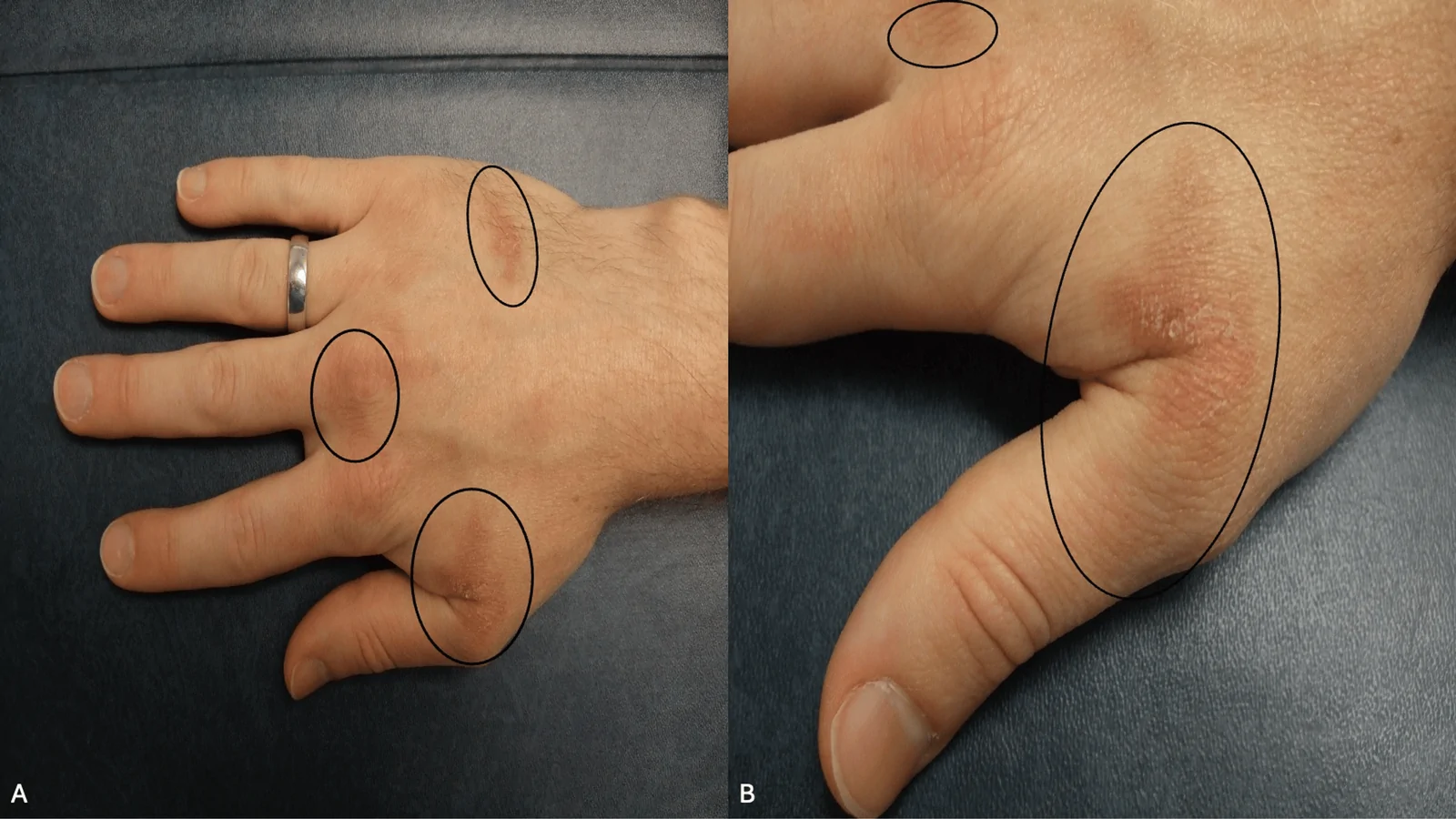
Classical presentation of margarita dermatitis on the dorsal right hand of a 29-year-old man Distant (A) and closer (B) views of the right dorsal hand of a left-handed 29-year-old man who had spent several hours outside on a porch when he was preparing (including the peeling and squeezing of fresh limes) and subsequently drinking margaritas. While he was outside, and exposed to the sun, he experienced the appearance of pruruitic and erythematous-based bullae. During the following two week, prior to visiting the dermatologist for an evaluation, the initial lesions progressively improved; at his appointment, there was hyperpigmentation with residual superficial desquamation at the sites of the prior blisters. The black ovals surround the residual dermatitis lesions and current hyperpigmented patches. The photograph in figure A has been republished with permission [9].

**Figure 9:**
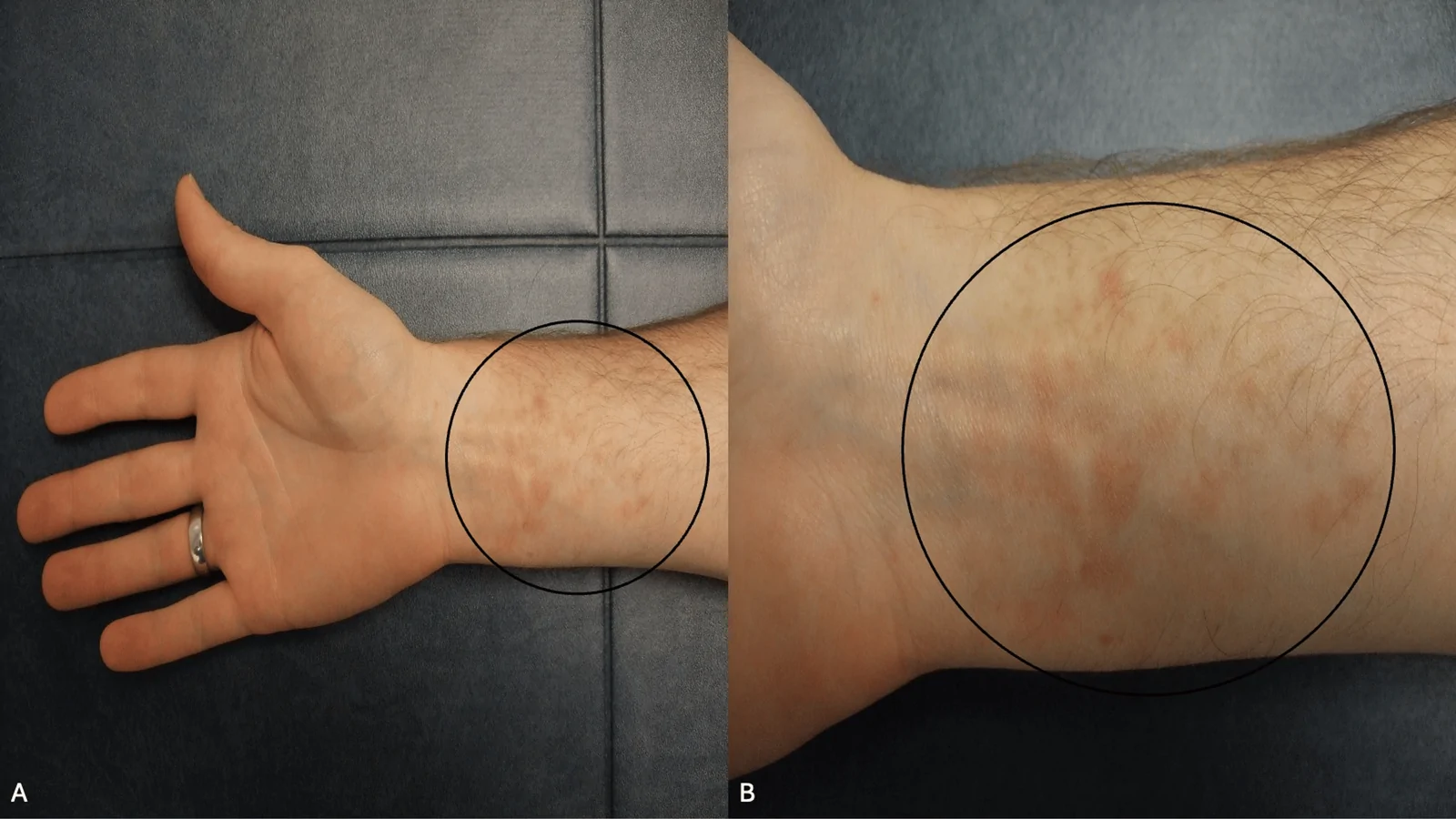
Classical presentation of lime-associated phytophotodermatitis caused by contact with lime and lime juice on the flexor wrist of the distal right upper extremity of a 29-year-old man Distant (A) and closer (B) views of the right flexor wrist of a left-handed 29-year-old man who had held the limes with the fingers of his right hand while cutting the citrus fruit using his left hand to hold the knife. Two weeks after the occurrence of acute lesions of margarita dermatitis, brown macules are present at the location of prior erythema or blisters or both on his right flexor wrist. The black oval surrounds the residual location of erythema and the current brown patches. The photograph in figure B has been republished with permission [9].

In some of the individuals, the onset of the acute lesion is delayed and may appear a few or several hours after sun exposure. Sometimes they appear the following day or even later. Less commonly, only delayed hyperpigmentation is the only manifestation of lime-associated phytophotodermatitis [6,10-13].

The clinician needs to maintain a high index of suspicion regarding the possibility of phytophotodermatitis. In many circumstances, the diagnosis is not considered when the patient is initially evaluated. Several different cutaneous diseases or systemic conditions have been postulated in individuals with phytophotodermatitis since the patient either does not remember their exposure to a causative exogenous agent or does not associate the relationship between the topical plant contact and subsequent sun exposure with the development of their condition [2,4-6]. The clinical differential diagnosis is summarized in Table 2 [3-6,9,10,17,18,23-26,29-31].

**Table 2:** Differential diagnosis of phytophotodermatitis ^a^The current table is a modification of the orginal table that has been republished with permission [9].

Category	Condition	Reference
Blistering dermatosis		[9,17,18]^a^
	Bullous pemphigoid	[9,18]^a^
	Porphyria cutanea tarda	[9,17,18]^a^
Dermal hemorrhage		[9,10]^a^
	Bruising (atypical)	[9,10]^a^
	Thrombocytopenia purpura	[9,10]^a^
Dermatitis		[3,5,6,9,10,17,18,23,25,29-31]^a^
	Allergic contact	[3,5,6,9,10,17,18,23,25,30,31]^a^
	Atopic	[6,9]^a^
	Irritant contact	[6,9,17,18,29]^a^
Infection		[5,6,9,10,17,18,23,25,30,31]^a^
	Cellulitis	[5,6,9,10,17,23,25,30]^a^
	*Erythema migrans *(Lyme disease)	[9,30,31]^a^
	Fungus (tinea)	[5,9,10,18,23,25]^a^
	Herpes simplex virus	[18]
	Herpes zoster virus (varicella zoster virus)	[9]^a^
	Impetigo (bullous)	[9,10,17,30,31]^a^
	Larva migrans	[9]^a^
	Lymphangitis (superficial)	[9,10]^a^
Medication-related		[3,9,10,18,26]^a^
	Drug-related photosensitivity	[3,18]
	Erythema multiforme	[9,10,18]^a^
	Fixed drug eruption	[9]^a^
	Stevens-Johnson syndrome	[26]
Other		[3,6,10,18,30,31]
	Jellyfish envenomation	[30,31]
	Pellegra	[18]
	Photoallergic reaction	[6]
	Solar erythema (sunburn)	[3,10]
	Solar urticaria	[18]
Pigmentary conditions		[9,18]^a^
	Melasma	[9,18]^a^
	Postinflammatory hyperpigmentation	[9,18]^a^
	Riehl melanosis	[9]^a^
Trauma		[3-6,9,10,17,18,24-26,30,31]^a^
	Chemical burns	[3,9,17]^a^
	Child abuse	[4,6,9,10,17,24,25,30,31]^a^
	Scalding	[6,9,17,24]^a^
	Thermal burn	[5,9,17,18]^a^

In some patients, the skin lesions are found at body sites that either the person’s hands touched themselves or locations that someone else, who had the lime juice on their hands, touched on the patient. Either handprints or fingerprints may be found. In some circumstances, these lesions have been misinterpreted to represent child abuse [6,8,10,28].

In contrast to the more common presentation of phytophotodermatitis, the condition can present as asymptomatic hyperpigmentation on the affected sun-exposed areas of the body. This has also been reported in several individuals with phytophotodermatitis that was associated with limes [6,10-13]. In addition, asymptomatic phytophotodermatitis presenting as hyperpigmented macules has also been reported in association with other citrus fruits, such as lemons, grapefruits, or oranges [10,13].

An 18-month-old Hispanic boy was evaluated for atypical bruising and possible malignancy. The boy’s mother described that the lesions initially appeared about six weeks earlier. The skin lesions presented as erythema that was predominantly at the locations where she held the toddler, such as his axillae, arms, and legs. The lesions were interpreted as bruises after they darkened; cutaneous examination revealed hyperpigmented bizarre lines, streaks, and macules on not only both axillae but also the boy’s four extremities. Additional history elicited during the dermatology evaluation revealed that the boy’s mother had been cutting and peeling lemons with her hands prior to the onset of the skin lesions and had picked her son after handling the lemons [10].

A 36-year-old right-handed Korean woman presented with sudden onset of several brown patches on the dorsal left hand; there was no preceding erythema or blistering. The prior week, she had been on a trip to California where she was sun-exposed outside for a large amount of time. She had used her bare hands to peel grapefruits and oranges. A biopsy of the pigmented area demonstrated hyperpigmentation of the basal layers of the skin and a slight increase in the number of melanocytes and orthokeratosis [13].

Some of the features of individuals with asymptomatic hyperpigmentation from lime-associated phytophotodermatitis, similar to the man described in this report, are summarized in Table 3 [6,10-13]. Lime-associated asymptomatic hyperpigmentation has been described in 14 individuals. The patients included 10 women and four men [6,10-13].

**Table 3:** Lime-associated phytophotodermatitis presenting as asymptomatic hyperpigmentation Abbreviations: C, case; CR, current report; d, days; F, female; L, left; M, male, R, right; Ref, reference; TI, time interval between exposure and manifestation onset; y, years; <, less than ^a^Several weeks is interpreted to be three to seven weeks: the asymptomatic lesions developed without any preceding erythema or vesiculation, several weeks after returning from a trip to Mexico [10]. ^b^An occupational photophotodermatitis or photoxicity on the dorsum of the hands of waiters who prepared cocktails with limes. It can occur in either amateur or professional bar staff who are exposed to sunlight after preparing mojitos. The investigators referred to the condition as acropigmentation of the dorsum of the hands from preparing mojitos: a lime-induced phytophotodermatosis [12]. ^c^A lesional biopsy of the hyperpigmentation showed orthokeratosis and slight increase in the number of basal melanocytes [13]. ^d^Shortly after is interpreted to be less than seven days: she noticed the area of discoloration shortly after a trip to Mexico [6].

C	Age (y)	Sex	Location	Morphology	Activity	TI (d)	Comment	Ref
1	2.5	M	Back, chest, right arm	Irregular patchy hyperpigmentation consistent with finger marks	Squeezing lime juice onto his mosquito bites	21-49^a^	Misinterpreted as thrombocytopenic purpura	[10] C7
2	6	F	Anterior chest, shoulders	Brown spots with bizarre pattern	Lime juice squeezed on site in outdoor hot spring water lime spa	2	Recent vacation in Fujian province; all lesions resolved in one month.	[11]
3	14	F	Dorsal hands	Irregular homogenous pigmentation	Making mojitos	14	Amateur bar staff^b^	[12] C8
4	16	F	Dorsal hands	Irregular homogenous pigmentation	Making mojitos	14	Amateur bar staff^b^	[12] C9
5	19	F	Dorsal hands	Irregular homogenous pigmentation	Making mojitos	21	Amateur bar staff^b^	[12] C4
6	21	F	Dorsal hands	Irregular homogenous pigmentation	Making mojitos	7	Amateur bar staff^b^	[12] C7
7	23	F	Dorsal hands	Irregular homogenous pigmentation	Making mojitos	7	Amateur bar staff^b^	[12] C1
8	25	F	Dorsal hands	Irregular homogenous pigmentation	Making mojitos	7	Amateur bar staff^b^	[12] C2
9	28	M	Dorsal hands and digits	Macular hyperpigmentation	Cutting and squeezing limes	3	Right handed, pigmentation completely gone by six months after onset	CR
10	30	F	Left dorsal hand, lateral fingers, and interdigital areas	Patches and partly reticulated irregular homogenous pigmentation	Squeezed limes with bare hands in cooking classes	7-10	Right handed, biopsy,^c^ during Thailand vacation while attending local cooking classes that used limes and on the beach a lot of the time	[13] C1
11	31	F	Dorsal hands	Irregular homogenous pigmentation	Making mojitos	7	Amateur bar staff^b^	[12] C3
12	34	F	Left inferior medial back	Irregular hyperpigmented patch	She touched her back with her left second, third, and fourth hand digits that were wet from a lime margarita	<7^d^	Beach vacation in Mexico	[6]
13	40	M	Dorsal hands	Irregular homogenous pigmentation	Making mojitos	10	Amateur bar staff^b^	[12] C6
14	41	M	Dorsal hands	Irregular homogenous pigmentation	Making mojitos	10	Amateur bar staff^b^	[12] C5

The patients ranged from 2.5 years of age to 41 years old (median, 23 years). The women ranged from six years to 34 years (median, 22 years). The men ranged from 2.5 years to 41 years (median, 34 years) [6,10-13].

The location of the asymptomatic hyperpigmentation was on the dorsal hands and digits in 11 individuals; one of these patients also had lesions on their interdigital areas. The remaining three patients without hand lesions each had pigmentation on their back. One of these three patients with back pigmentation also had chest pigmentation, and another had pigmentation on both his chest and right arm [6,10-13].

Similar to the patient in this report, the clinical presentation was the sudden and unexpected appearance of asymptomatic irregular patches of pigmentation; the pattern of the darkened skin was typically homogeneous and frequently irregular in pattern. Occasionally (in two patients), the patches of hyperpigmentation were consistent with finger marks, and rarely (in one patient) it was reticulated [6,10-13].

The lime contact prior to sun exposure was most commonly in the preparation of an item that was intended for consumption as either a beverage to be drunk or a meal to be eaten [12,13]. Nine of the patients were preparing mojitos as bar staff or waiters, and the reported patient was slicing and securing juice from limes to be used in margaritas [12]. One woman had attended a local cooking class in Thailand during which she squeezed limes with her bare hands during the food preparation [13].

The exposure to lime was unintentional in one woman. Three of the fingers on her left hand had become wet from a margarita she was drinking. She wiped the wet fingers (with the lime-containing margarita beverage) on her back [6].

A 2½-year-old Hispanic toddler had been on a trip to Mexico and decided to squeeze lime juice onto the mosquito bites that were located on his chest. Some of his patchy hyperpigmentation looked like finger marks. When the boy developed a fever, the pediatricians at the Children’s Hospital outpatient clinic diagnosed him with an otitis media ear infection and misdiagnosed his pigmented chest lesions as thrombocytopenic purpura [10].

An Asian six-year-old girl from Beijing, China, was on a holiday in the Fujian province. She and her father visited an outdoor hot spring water lime spa. Forty-eight hours after she squeezed the lime juice onto her shoulders, asymptomatic brown macules appeared on her back and anterior chest [11].

The time interval from lime exposure to onset of hyperpigmentation ranged from days (two) to weeks (three to seven); the median was seven days. The asymptomatic pigmentation appeared in a week or less after contact with the lime juice and subsequent exposure to the sun in 57% of the individuals (eight of the 14 patients). In only 28.6% (four of the 14 patients), the hyperpigmentation initially presented at or after two weeks from the lime contact [6,10-13].

The nomenclature of phytophotodermatitis related to limes is listed in Table 4 [1,4-13,17,23,25-35]. The diagnosis of margarita photodermatitis was introduced by Abramowitz et al. in March 1993 in a letter to the editor that described a 30-year-old man who developed burning, severe swelling, and blistering of his left hand after he had squeezed five dozen limes while preparing margaritas. The remainder of the afternoon, after he had completed the preparation of a large quantity of the beverage, was spent sunbathing [1].

**Table 4:** Nomenclature of phytophotodermatitis related to limes ^a^The authors introduced a new acrostic: MAgarita DErmatitis by a Sliced/Squeezed Lime-Associated Phytophotodermatitis (MADE by a SLAP). MADE by a SLAP includes the first two letters of two of the words margarita and dermatitis: MA for Margarita and DE for Dermatitis. The two words “by a” follow MADE. The second half of the acrostic is created by incorporating the first letter of the following words: Sliced/Squeezed, Lime, Associated, and Phytophotodermatitis; “S” for sliced or squeezed, “L” for lime, “A” for associated, and “P” for phytophotodermatitis. The acronym incorporates the fruit related to the skin condition (lime), the activity involving the fruit that resulted in exposure to the fruit juice (slicing and squeezing the fruit), the pathogenesis of the cutaneous disorder (a phytophotodermatitis), the alcoholic beverage being prepared (margarita), and the skin condition (dermatitis). The phrase “MADE by a SLAP” assists in describing the morphology of the erythema and hyperpigmentation that may be observed at the locations on the body that are touched by the hands.

General name	Specific name	Year	Reference
Food-associated dermatitis			[3,32,33]
	Guacamole dermatitis	2017	[32,33]
	Thai dermatitis	2018	[3]
Lime dermatitis			[28]
	Lime dermatitis	2024	[28]
Lime disease			[7,10,28]
	Endemic “lime” disease	1994	[10]
	Lime disease	2022	[7]
	The other “lime” disease	1999	[28]
Lime-associated			Current report
	Sliced/squeezed lime-associated phytophotodermatitis (SLAP)^a^	2026	Current report
Lime-induced phytophotodermatitis			[2,5,11,25]
	Lime-induced phytophotodermatitis	2014	[25]
	Lime-induced phytophotodermatitis	2019	[5]
	Lime-induced phytophotodermatitis	2020	[11]
	Lime-induced phytophotodermatitis	2024	[2]
Margarita associated			[1,3,6-9] Current report
	Margarita burn	2021	[3]
	Margarita dermatitis (phytophotodermatitis)	2009	[9]
	Margarita dermatitis (MADE)^a^	2026	Current report
	Margarita dermatitis	2022	[7]
	Margarita photodermatitis	1993	[1]
	Margarita photodermatitis	2025	[8]
	Phytophotodermatitis from lime margaritas	2024	[6]
Other alcoholic beverage associated			[12,26,30,31,34]
	Acrodermatitis of the dorsum of the hands from preparing mojitos: a lime-induced phytophotodermatosis	2016	[12]
	Corona, lime, sun, rash: a case of severe phytophotodermatitis in an active duty soldier	2023	[26]
	Lime, beer and phytophotodermatitis	2012	[31]
	Mexican beer dermatitis: a unique variant of lime phytophotodermatitis attributable to contemporary beer-drinking practices	2010	[30]
	Mexican beer hand	2023	[26]
	Mojito-induced phytophotodermatitis: a case of lime-triggered skin reaction	2025	[34]
Phytophotodermatitis from limes			[17,29,32,33,35]
	Chronic phytophotodermatitis from limes	2021	[17]
	Occupational confetti-like phytophotodermatitis due to lime fruit	2022	[29]
	Phytophotodermatitis	2017	[33]
	Phytophotodermatitis and limes	2007	[35]
	Phytophotodermatitis from lime juice	2017	[32]
Phototoxic dermatitis to limes			[27]
	Pomander dermatitis	1987	[27]

In May 1994, a group of investigators reported 10 children with phytophotodermatitis whom they evaluated during a 15-month period and who had all been misdiagnosed. The children were from San Diego, California. The children had been referred to the pediatric dermatology service with incorrect diagnoses. However, during their clinical evaluation, the investigator realized that the lesion morphologies on the children were distinctive; they included bizarre irregular sunburns, drip marks, hand-print shapes, and hyperpigmented streaks. Importantly, based on the presentation of the skin lesion configurations, a history of contact with either limes or lemons and subsequent sun exposure was able to be elicited. The investigators referred to the condition as endemic “lime” disease in their publication that described the phytophotodermatitis that had occurred in San Diego County [10].

In 1999, the condition was described as the other “lime” disease in a 27-year-old who frantically presented to an emergency department in San Antonio, Texas, with a new skin condition that she had never previously experienced. Her upper and lower extremities both had an eruption of blisters; in addition, her right lateral thigh showed hyperpigmented parallel linear streaking. The clinicians initially interpreted her situation to be an allergic reaction and provided symptomatic treatment (with systemic diphenhydramine hydrochloride) and a referral to the dermatologist. Direct questioning during her dermatology appointment revealed that she had been vacationing in Mexico, where she had been squeezing limes for beverages that she was drinking on the beach; since she did not have a knife, the limes were torn open using her hands. Two days after returning from her vacation, the phototoxic eruption appeared; the parallel thigh streaks correlated with her wiping excess lime juice from her fingers on her proximal lower extremities. The designation of the woman’s condition as the other “lime” disease by the researchers was to emphasize its difference from the homophone “Lyme” disease (a tick-borne condition that originated in Lyme, Connecticut); the pronunciation of both words (lime and Lyme) is similar, yet their spelling and meaning are different [4].

The condition was recommended to be referred to as margarita dermatitis by one of the authors (PRC) in 2015 when it was associated with the preparation of margaritas [9]. A 29-year-old man developed red-based itchy blisters on his right dorsal hand and flexor wrist; the symptoms and skin lesions appeared after he had sliced and squeezed fresh limes into the margaritas he was preparing and drinking over several hours outside on a sun-exposed porch [19]. Other researchers also favored including the word margarita in the name of the condition when the beverage was related to the development of the dermatosis [1,3,6-9].

When the dermatitis was related to other alcoholic beverages, the name often incorporated the etiologic agent in the diagnosis. When the condition followed the consumption of beer, it was referred to as either Mexican beer dermatitis or Mexican beer hand [26,30]. It was also designated as either ‘Corona, lime, sun, rash’ or ‘Lime, beer and phytophotodermatitis’ [26,31].

Mojito is an alcoholic Cuban drink that was created in the United States during the Prohibition era. Lime is a component of a mojito; in addition, this beverage also consists of mint, rum, sparkling water, and sugar [12,17,34]. A group of investigators observed nine waiters who developed occupational phytophotodermatitis after preparing cocktails that included limes; the phytophotodermatitis occurred in amateur or professional bar staff whose hands were exposed to sunlight after preparing mojitos [12].

Another researcher described a 25-year-old woman who was on a summer vacation and accidentally spilt a mojito cocktail containing lime on her left forearm; the area had prolonged exposure to the sun. The patient initially developed mild discomfort and a burning sensation on her forearm. A linear, irregular erythematous plaque covered with small vesicles appeared on her left forearm. The erythema and vesicles gradually healed over the next few days, and she noticed a striking, linear, hyperpigmented lesion on her left upper extremity that followed the area of contact where the lime juice had spilt [34].

Limes have been used for the preparation of a dip or a meal. We suggest that the name of the lime-related phytophotodermatitis be associated with the specific food [13,32,33]. A right-handed 30-year-old woman developed asymptomatic hyperpigmentation after participating in cooking classes in Thailand in which she squeezed limes with her bare hands. She developed Thai dermatitis on the left dorsal hand, lateral fingers, and interdigital areas [13].

Guacamole is a creamy dip or sauce whose main component is mashed green avocados, lime juice, and salt; other optional components include chillies, cilantro, onions, and tomatoes. Guacamole dermatitis occurred in two women [32,33]. The first was a 22-year-old female college student with a painful erythematous blistering rash demonstrating a splash pattern on her left upper extremity who had been squeezing limes into guacamole prior to spending several hours sunbathing [33]. The second was a 36-year-old pregnant woman with an uncomfortable blistering eruption involving both of her hands after making guacamole at an outdoor picnic [32].

An outbreak of phototoxic dermatitis due to the Persian (common) lime occurred in the summer of 1984 in a day camp in a suburb of Baltimore, Maryland. A lime-associated condition occurred in these campers and counselors who were making pomander balls, a form of sachet. We suggest referring to the lime-related phytophotodermatitis in these individuals as pomander dermatitis [27].

Pomander dermatitis occurred in 97 of 622 (16%) of the examined children and in seven (7%) of the examined counselors. A pomander ball was created by using scissors to puncture the lime peel, which results in lime peel and juice contacting the hands; thereafter, cloves were stuffed into the holes. The lime is then rolled in cinnamon prior to being wrapped in cloth. The body sites affected by pomander dermatitis included the dorsal hand, extensor and flexor wrists and forearms, and the webbed area between the thumb and forefinger [27].

In this report, we introduce a new acrostic: *MA*garita *DE*rmatitis by a *S*liced/*S*queezed *L*ime-*A*ssociated *P*hytophotodermatitis (*MADE* by a *SLAP*). MADE by a SLAP includes the first two letters of the words margarita and dermatitis: “MA” for margarita and “DE” for dermatitis. The two words “by a” follow MADE. The second half of the acrostic is created by incorporating the first letter of the following words: sliced/squeezed, lime, associated, and phytophotodermatitis; “S” for sliced or squeezed, “L” for lime, “A” for associated, and “P” for phytophotodermatitis. The acronym incorporates the fruit related to the skin condition (lime), the activity involving the fruit that resulted in exposure to the fruit juice (slicing and squeezing the fruit), the pathogenesis of the cutaneous disorder (a phytophotodermatitis), the alcoholic beverage being prepared (margarita), and the skin condition (dermatitis). The phrase “MADE by a SLAP” assists in describing the morphology of the erythema and hyperpigmentation that may be observed at the locations on the body that are touched by the hands after contact with the lime and prior to sun exposure.

## Conclusions

Margarita dermatitis is a phytophotodermatitis that occurs after topical contact with either lime peel or lime juice, or both, with the skin and subsequent sun exposure to the lime-contacted skin. Mojito dermatitis and Mexican beer dermatitis are other alcoholic beverage-related variants of this lime-associated phototoxic skin reaction. After the skin that has been in contact with the lime is exposed to the sun, the acute onset of symptoms and lesions of lime-induced phytophotodermatitis, such as painful and occasionally pruritic erythema and blisters, promptly occurs. Postinflammatory hyperpigmentation usually occurs as the initial lesions resolve; the pigment changes often eventually improve or completely resolve. The patient is frequently unaware that lime contact with the skin followed by exposure to the sun resulted in their new cutaneous lesions developing. In addition, the individual evaluating these patients often does not make the association; therefore, the clinician needs to have a high index of suspicion that the new skin lesions may represent a phytophotodermatitis and obtain an appropriately relevant history from the patient. Asymptomatic delayed hyperpigmentation is an infrequent presentation of lime-related phytophotodermatitis. Margarita dermatitis presented with asymptomatic pigmentation on the hands of a 28-year-old man three days after exposure to limes and sunlight. We introduce a new acrostic for phytophotodermatitis caused by lime-related margarita dermatitis: *MADE* by a *SLAP* (*MA*garita *DE*rmatitis by a *S*liced/*S*queezed *L*ime-*A*ssociated *P*hytophotodermatitis).
